# Could long-term overhead load in painters be associated with rotator cuff lesions? A pilot study

**DOI:** 10.1371/journal.pone.0213824

**Published:** 2019-03-15

**Authors:** Markus Loew, Sepehr Doustdar, Christoph Drath, Marc-André Weber, Thomas Bruckner, Felix Porschke, Patric Raiss, Marcus Schiltenwolf, Haidara Almansour, Michael Akbar

**Affiliations:** 1 German Joint Center, Atos Clinic, Heidelberg, Germany; 2 Department of Orthopaedic Surgery, Trauma Surgery and Spinal Cord Injury, University of Heidelberg, Heidelberg, Germany; 3 Occupational Medicine And Safety Service Employer’s Liability Insurance Association For The Construction Industry, Heidelberg, Germany; 4 Department Of Diagnostic And Interventional Radiology, Heidelberg University Hospital Heidelberg, Germany; 5 Institute of Medical Biometry and Informatics, University of Heidelberg, Heidelberg, Germany; 6 BG Trauma Center Ludwigshafen, Ludwigshafen, Germany; 7 Orthopedic Clinic in Munich, Munich, Germany; Augusta University, UNITED STATES

## Abstract

**Background:**

Use of the arm above shoulder level has been described as a risk factor for developing rotator cuff tears (RCT). There is a lack of information regarding the frequency and distribution of RCT in a population using their arms above shoulder level during daily work. The aim of this study was to analyze clinical and radiographic findings in a population of painters working more than 10 years and to compare the results with a control group (CG)

**Materials and methods:**

100 individuals working more than 10 years as a painter were compared to 100 matched controls without using their arms above shoulder level. MRI scans were performed in all participants. Clinically, the Constant score, DASH score and range of motion (ROM) of the shoulders were analyzed.

**Results:**

In the painter group (PG) a tear of the supraspinatus tendon was detected in 45% (10%full-thickness; 35% partial) compared to 8% in the (CG) (3% full-thickness; 5%partial; p<0.001). Impingement test was painful in 52% of PG and 7% of CG (p<0.001). The Constant score in the PG was significantly lower compared to the CG (62 vs. 93 points; p<0.001). PG had significantly worse DASH scores and inferior active and passive ROM of the dominant shoulders. In the multivariable logistic regression analysis, three risk factors (smoking, sports activity, and handedness) showed no effect. However, group membership (PG/CG) and age revealed a significant effect.

**Conclusion:**

Long-term occupational load on the shoulders in painters seems to be associated with an increased risk for developing RCT. Our findings may provide support for developing preventive strategies for this unique cohort.

Level of evidence: III

## Introduction

Rotator cuff tears (RCT) of the shoulder are tendon injuries that, from the sixth decade of life onward, are associated relatively often with degenerative changes and are less frequently the result of trauma.

Symptoms of chronic RCT are pain, weakness, and a restricted range of motion [1]. At younger ages, degenerative RCT are relatively uncommon. The tendon of the supraspinatus muscle is affected most often. There is no consensus on the extent to which overuse or pathological loading of the rotator cuff (RC) causes structural damage. RCT have often been described in athletes after long-term participation in throwing disciplines [2] but these are of a different location and type than that of degenerative defects. Biomechanical and kinematic studies have shown that strain on the supraspinatus tendon is greatest at around 35° abduction of the arm [3–8]. It is postulated that working with the arm held at and above shoulder level causes impingement, with compression of the supraspinatus tendon under thecoraco-acromial arch, potentially leading to tendon injury through mechanical wear and ischemia [9–12].

Moreover, electromyographic studies have demonstrated that shoulder muscle activity is increased by forced gripping movements of the hand [13]. Various epidemiological investigations have revealed that manual workers who perform repetitive tasks with the arm held above shoulder level are more frequently afflicted by symptoms in the shoulder and neck [14–17]. Data in these studies were mostly acquired by means of specific questionnaires and clinical examination [18, 19]. They show a stress-associated increase in symptom complexes described as impingement syndrome [20].

Little is known on whether long-term occupational stress with the arm in positions between hip and shoulder level also results in structural damage to the tendons [9]. In Germany, between 2012 and 2015 about 127.000 to 130.000 painters were employed [21]. Work place analysis of painters showed: manual activities of painters include more than 75% plastering, painting with brush or roller and wallpapering. Furthermore, It is known that those manual workers are more at risk to develop shoulder pain (symptoms) and alteration [9].

We therefore designed a cross-sectional study to test the hypothesis that working as a painter for a period of at least 10 years is associated with an RC tear.

## Materials and methods

All painting companies in the Rhein-Neckar region of the federal state of Baden-Württemberg in Germany were contacted. Full-time painters who had been working for at least 10 years without interruption were included.

All participants provided their written informed consent before initiation of the study. IRB approved our study. Ethics Committee of the faculty of medicine at Heidelberg University granted permission no. S 261–2008.

Exclusion criteria were previous shoulder injuries or operations, previous cervical injuries, operations or any kind of treatment, relevant neurological and rheumatic diseases, regular participation in sports involving hitting/throwing a ball or stressing the arms above the head (e.g. tennis and handball, basketball), and employees claiming workers’ compensation.

Recruitment was ended on reaching the planned painter group (PG) size of 100 subjects.

The control group (CG), for which the same exclusion criteria applied, consisted of 100 age-and sex-matched persons who had responded to an advertisement in the regional press. In the advertisement, the inclusion and exclusion criteria were defined in detail. The CG contained a representative assortment of occupations. Participants who were taking part of overhead sports on a regular basis (once a week) or who were involved in activities which involved overhead work were excluded. Volunteers with a work place analysis which revealed repetitive manual work in shoulder height or above shoulder level were also excluded

Each participant underwent a physical examination. The range of active and passive motion of the shoulder joints were measured using a goniometer. Goniometer measurements as well as clinical tests were conducted once by an orthopaedic surgeon with a fellowship in shoulder surgery. Any signs of impingement (Neer- and Hawkins-Test) were recorded, as were the results of the provocation tests for the RC (the Jobe test for the supraspinatus muscle, external rotation stress for the infraspinatus muscle, the lift-off test for the subscapularis muscle, and the palm-up test for the long biceps tendon [22]).We chose these tests to see if movements above the shoulder level were painful and if there was pain under loading or motion. Symptoms and shoulder function were assessed by Constant and Murley score [23] and the DASH (Disability of Hand, Arm, and Shoulder) score [24]. The DASH scoring system assesses the ability to perform various activities using the upper limb, with a lower score indicating greater ability. Both shoulders of all study subjects were examined using 3 Tesla MRI scanner (Magnetom VerioTM; Siemens, Germany) according to a standard protocol (sagittal, coronal, and axial planes in 3-mm slices, comprising T2-weighted TSE sequences, PD-weighted TSE sequences, and T1-weighted SE sequences). Images were assessed by three blinded radiologists who were fellowship trained in musculoskeletal radiology. The tendons of the supraspinatus, infraspinatus, and subscapularis muscles were analyzed on MRI and classified as intact, partial tear (articular, bursal, or intratendinous), or complete tear [25].

### Statistical methods

Continuous data was reported as mean and standard deviation, in the case of categorical variables as absolute and relative frequencies. Possible differences between groups were evaluated by means of the Wilcoxon U-test for continuous data and the chi-square test for categorical data. Multivariable logistic regression analysis was used to detect parameters that might influence the binary primary endpoint (RCT yes/no) or binary secondary endpoints. Odds ratios with 95% confidence intervals were calculated to quantify the risk of outcomes between groups. Sample size was set at 100 per group. The sample size could not be formally calculated due to missing information about the primary endpoint, therefore this pilot study was undertaken to decide how and whether to launch a full-scale project. The level of significance α was set at 5%.

## Results

All members of both groups were males because the painting firms had no female employees who fulfilled the inclusion criteria. None of the painters who were approached declined to participate. 17 painters were found ineligible due to prior shoulder surgery. Epidemiological characteristics of the participants—age, handedness, sporting activities, and other relevant characteristics and activities—are listed in Table 1.

**Table 1:** Demographic characteristics of the painters’ group and the control group.

Characteristic	Painters’ group	Control group
Age (years)	46 ±9	46 ±9
Height (cm)	177 ±7	178 ±6
Weight (kg)	83.1 ± 8.8	82.3 ±10.8
Body mass index	26.5 ±2.4	26 ±2.8
Right handedness	92%	79%
Recreational sport	25%	70%
Regular alcohol consumption	9%	10%
Smokers	66%	28%

Painters worked 63.6% (Table 2) of their time at shoulder level or above shoulder level.

**Table 2:** Relevant details of occupational history in the painter group and control group.

	PG	CG
Years worked (mean +/- SD)	21 ± 7.2	20.7 ± 8.9
Years worked (minimum / maximum)	10 / 41	3 / 41
Hours of work / week (mean +/- SD)	41 ± 3.9	37.3 ± 6.7
Manual activities (plastering, painting with brush or roller, wallpapering)	75.3%	-
Proportion of time working with hands above hip level	63.6%	-
Lifting of heavy weights (5–25 kg) in seconds / hour of work	41	-

The parameters quantifying the stress during working time are taken from a representative study carried out by the occupational medicine and safety service of the Employer's Liability Insurance Association for the Building Industry (*Berufsgenossenschaft der Bauwirtschaft*).

PG, Painter group; CG, Control group; SD, Standard Deviation

The work place analysis of the CG revealed that most of them were working in an office.

On average, members of PG had been working in their occupation for over 21years. Table 2 shows details of the occupational stress profile in the PG and CG. All study subjects completed a questionnaire concerning the presence of previous or current treatment of shoulder symptoms.

Functional symptoms were reported such as pain, restricted active ROM, (Table 3) and reduced strength. Active motion in the PG was restricted in all degrees of freedom compared with the CG. In the PG, 82% of the study subjects reported previous or current shoulder symptoms, restricted to the dominant side in 27% and bilateral in 48% of cases. The equivalent figure in the CG was 25%, 10% on the dominant side and 10% bilateral.

**Table 3:** Difference of angles and pain in Subjects without (n = 139) and with (n = 61) rotator cuff tear, dominant side.

Variable	Mean difference (95% CI)
Anteversion	-48.7 (-61.7;-35.6)
Retroversion	-7.7 (-9.7;-5.6)
Abduction	-61.1 (-75.2;-47.1)
Adduction	-7.8 (-9.8;-5.7)
External rotation (ERO)	-10.5 (-15.3;-5.8)
Internal rotation (IRO)	-16.5 (-22.4;-10.5)
ERO in 90° Abduction	-36.4 (-46.2;-26.5)
IRO in 90° Abduction	-37.3 (-47.1;-27.6)
Pain score(Constant Score)	7.7 (6.6;8.9)

CI, Confidence Interval. Spearman‘s rank correlation between angles and pain score (Constant score) ranged from -0.53 to -0.69. PG and CG with a rotator cuff tear and pain had less range of motion (high negative correlation).

Symptoms had persisted for 5 years or longer in 40% of the PG and 6% of the CG. Altogether, 55% of the PG and 8% of the CG had received analgesic treatment and physiotherapy for shoulder problems. Correlation of pain score (numerical rating scale, part of the Constant score) and the range of motion revealed a strong negative relationship between pain and rage of motion when analyzing the total sample (Table 3).

Furthermore, movement was painful in more than half of the PG (Table 4). Impingement signs and provocation tests were pathologic significantly more often in the PG for the supraspinatus muscle.

**Table 4:** Active range of motion and pain caused by moving the dominant shoulder joint.

	Painters’ group	Control group
Anteversion (mean +/- SD)	137.5 ±46.3	169.5 ±2.6
Anteversion painful	53 (53.0%)	6 (6%)
Abduction (mean +/- SD)	137.9 ±52	177.9 ± 4.1
Abduction painful	58 (58%)	8 (8%)
External rotation in 90° abduction (mean +/- SD)	56.4 ± 35.1	80.1 ± 0.5
External rotation painful	37 (37%)	3 (3%)

SD, Standard Deviation

**Fig 1:**
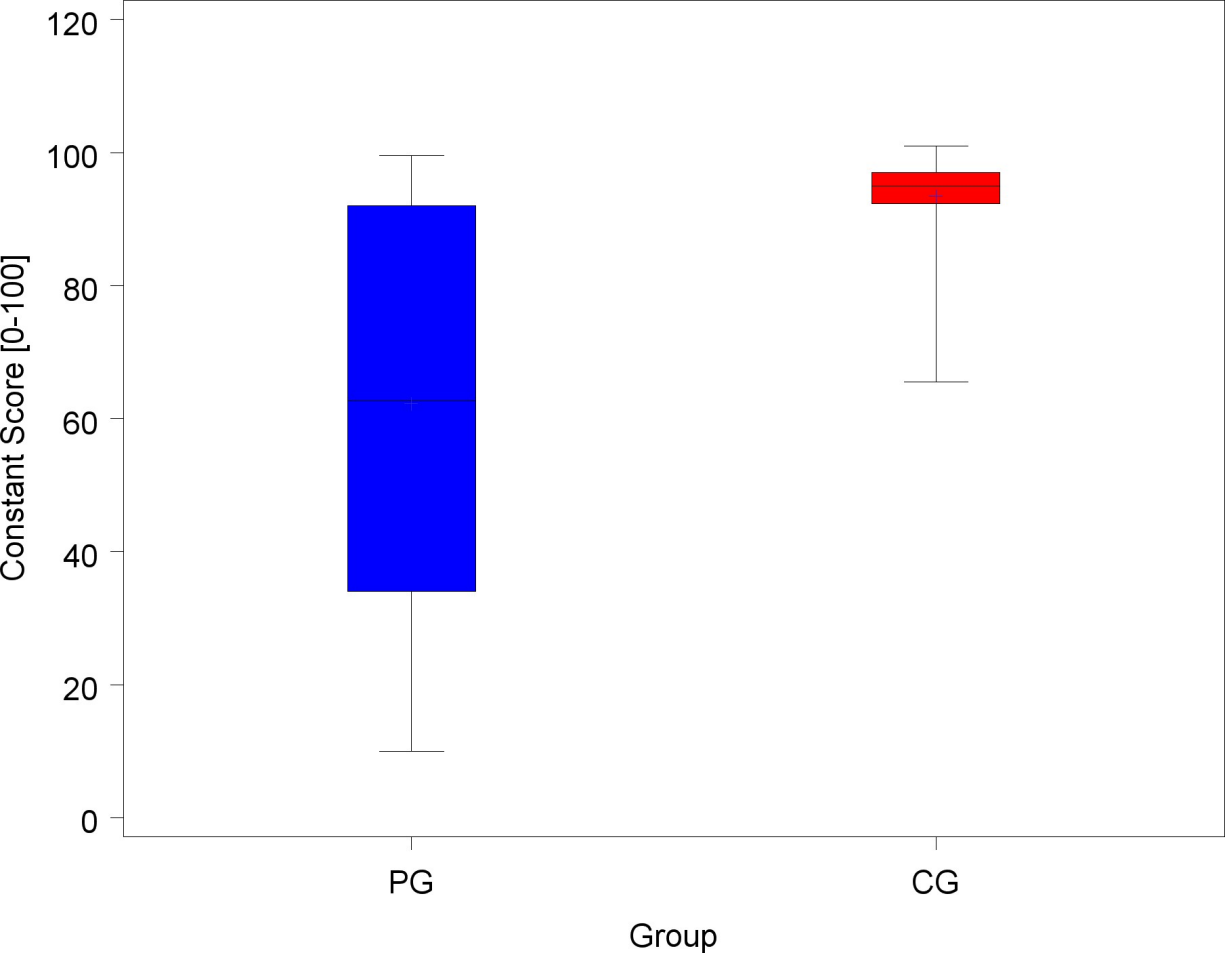
Box plots of the Constant score for shoulder joint on the dominant side (PG; painters’ group; CG, control group). The mean DASH scores were 25.7 (± 17.5) for the 124 PG and 4 (± 8.9) for the CG (p <0.001).

Table 5 illustrates the results of impingement and provocation tests on the dominant side. PG also fared significantly worse on all scales of the Constant and Murley score (Table 6; Fig 1).

**Table 5:** Results of impingement tests and provocation tests for the shoulder joint on the dominant side.

Test	Painters’ group	Control group
Impingement (Neer) positive	52 (52%)	7 (7%)
Impingement (Hawkins) positive	43 (43%)	4 (4%)
Supraspinatus (Jobe) positive	54 (54%)	5 (5%)
Infraspinatus (ARO stress) positive	19 (9%)	0 (0%)
Subscapularis (lift-off) positive	6 (6%)	0 (0%)
Biceps (palm-up) positive	45 (45%)	2 (2%)

**Table 6:** Constant score for the shoulder joint on the dominant side (scales: pain, activities of daily life, pain-free motion, strength).

Scale	Painters’ group	Control group
Pain (points) (median +/- SD)	7.5 ±5.3	13.6 ± 2.6
Activity (points) (median +/- SD)	12.2 ± 5.9	19.3 ± 2
Motion (points) (median +/- SD)	27± 11.8	38.9 ± 2.4
Strength (points) (median +/- SD)	15.8 ± 9	21.6 ± 3.1
Total (median +/- SD)	62.3 ± 29.6	93.4 ± 7.2

SD, Standard Deviation

### MRI analysis

MRI Findings are shown in Table 7. The overall agreement between the three blinded radiologists was very good with a kappa ranging from 0.82 to 0.94. Analysis revealed a strong association between the diagnosis of RCT and PG (OR = 11.3; 95%-CI 4.868–26.182)). In the majority of cases, the tendon affected was the supraspinatus tendon, which showed a partial tear in 35% (p < .0001) and a complete tear in 10% (p = 0.045) of the PG (Table 7). The articular (p < .0001) and the bursal (p = 0.0094) partial tears of supraspinatus tendon were significantly higher in the PG. Complete tear (p = 0.045) of supraspinatus tendon was significantly higher in the PG.

**Table 7:** Rotator cuff tears as detected by MRI.

Tendon			Painters’ group	Control group
Supraspinatus	Intact		55	92
	Partial tear		35 (20/9/6)	5
		Articular	20	2
		Bursal	9	1
		Intratendinous	6	2
	Complete tear		10	3
Infraspinatus	Intact		98	99
	Partial tear		2	1
	Complete tear		0	0
Subscapularis	Intact		91	99
	Partial tear		8	1
	Complete tear		1	0
Total			52	9

Table 8. descriptively summarizes other shoulder pathologies as found in the MRI such as tendinopathies, bursitis and arthrosis of the acromio-clavicular joint.

**Table 8:** Summary of other shoulder pathologies as found in the MRI.

Rotator cuff tendinopathies				
Localisation	PG		CG	
	No.	%	No.	%
Supraspinatus	65	65	43	43
Subscapularis	14	14	4	4
Infraspinatus	4	4	0	0
Teres minor	1	1	0	0
**Total**	**67**	**67**	**44**	**44**
**Biceps tendon injury**				
**Finding**	**PG**		**CG**	
	**No.**	**%**	**No.**	**%**
Tendinopathy	27	27	14	14
Luxation	2	2	0	0
Rupture	1	1	0	0
**Bursitis**				
**Localization**	**PG**		**CG**	
	**No.**	**%**	**No.**	**%**
Bursitis subacrominalis	13	13	5	5
Bursitis subdeltoidea	7	7	6	6
**Acromio-clavicular joint arthrosis**				
**PG**			**CG**	
**No.**	**%**		**No.**	**%**
39	39		29	29

Multivariable logistic regression analysis was used to evaluate the influence of group membership (PG/CG), smoking status (yes/no), sports activity (yes/no), handedness (right/ left), and age on the presence of RCT. Only group membership (PG/CG) and age showed an effect in the multivariable logistic regression (Table 9).

**Table 9:** Odds Ratio of rotator cuff tear (RCT) with the co-variables smoking, sports, handedness and age: Results of logistic regression analysis. Sensitivity analysis with different logistic regression models showed similar results.

Variable	RCT		Odds ratio Multivariable (95% CI)	p-value
	Yes	No		
Group - PG	66 (80.5%)	34 (28.8%)	11.289 (4.868–26.182)	< .0001*
Group - CG	16 (19.5%)	84 (71.2%)		
Smoking- Yes	49 (59.8%)	45 (38.1%)	1.257 (0.608–2.596)	0.537
Smoking - No	33 (40.2%)	73 (61.9%)		
Sport -Yes	24 (29.3%)	71(60.2%)	0.88 (0.394–1.998)	0.773
Sport - No	33 (40.2%)	73 (61.9%)		
Right-handed	71 (86.6%)	100 (84.8%)	1.878 (0.703–5.014)	0.209
Left-handed	11 (13.4%)	18 (15.3%)		
Age	47.5±8.2	44.9±9.4	1.052 (1.007–1.009)	0.024

*, significant; CI, Confidence Interval; R, Right; L, Left

## Discussion

This cross-sectional study of a representative group of male persons residing in Germany revealed that one quarter of the population had suffered symptoms in the dominant shoulder by the age of 50 years; 8% had sought medical assistance for this reason. 7% showed symptoms of impingement at clinical examination, and the average Constant score was 93 points. MRI demonstrated complete RCT in 3% and partial RCT in 6% of this group.

In contrast, symptoms in the dominant shoulder had been or were currently being experienced by 75% of an age-matched group of men who were professionally working as painters for at least 10 years (average 21 years) and regularly held their arms above shoulder level in the course of their work. More than half of these workers had received medical treatment for this reason.

The painters’ active and passive shoulder joint mobility was restricted in all degrees of freedom compared with the normal population, and more than half of them experienced pain. Half of the painters presented with symptoms of impingement, and their Constant score—overall and on all subscales—was significantly lower than in the CG. MRI showed complete RCT in10% of the painters and partial RCT in 35%. This study showed that the incidence of RCT in PG was significantly higher than in the normal population. An association between the occurrence of RCT and long-term, regular stress on the arm in positions above shoulder level is therefore probable. It is worth mentioning, that PG and CG were excluded when there was a history of weight lifting and any kind of overhead sports activities.

### Etiology and risk factors for RCT in the setting of work-related injury

The etiology of RCT is multifactorial: not only extrinsic factors such as impingement [2, 20, 26], shape [27, 28], and lateral extension of the acromion [29] play a part, but also genetic and intrinsic factors such as perfusion-related changes and other tendinopathies. Workers who regularly hold their arms in an elevated position have a high rate of shoulder symptoms [12]. Silverstein et al. [15] investigated 733 workers in 12 workplaces who were exposed to various chronic overhead stresses. RC syndrome was reported in 7.5% of cases. A significant risk factor was performing long, monotonous tasks with the arms in more than 45° flexion. Other authors have made similar observations [10, 30]. Data on structural lesions of the RC in skilled workers are sparse. Svendsen et al. [10] studied 136 machinists, auto mechanics, and painters aged between 40 and 50 years who had practiced their occupation for at least 10 years. MRI of the dominant right shoulder revealed focal degeneration and partial tears of the supraspinatus tendon in 35.3% of cases, with complete tears in 2.9%. The authors concluded that the RC disorders were work related.

### Age, comorbidities, smoking and group membership (PG vs, CG) in the context of RCT

In all other studies we could find, changes were documented in the RC from the age of 50 years upward. Tempelhof et al. [31] examined asymptomatic subjects and found RCT in 13% of those in the sixth decade and 20% in the seventh decade of life. Moosmayer et al. [29] investigated asymptomatic volunteers. They found full-thickness tears in 2.1% of those between 50 and 59 years, and for the age group 60 to 69 the rate was 5.7%. Fehringer et al. [32] reported a prevalence of 22% in a cohort of patients aged 65 and over who were receiving inpatient treatment for unrelated internal medical conditions and correlated the US findings with symptoms, shoulder function, and co-morbidities. Based on the results of Fehringer et al., which found no significant correlations between co-morbidities and RCT, we did not include any co-morbidities in this study. Other researchers have discussed the association of smoking and overweight, with the occurrence of RCT. Particularly for smoking the findings are inconsistent. Baumgarten et al. [33] described a duration- and dose-dependent correlation between cigarette smoking and RCT. Of the 586 patients, 375 were smokers and 211 nonsmokers; authors calculated an elevated risk of RCT for those with a positive smoking history (61.9% vs. 48.3%). The odds ratio (OR) for occurrence of RCT was calculated as 1.74 for smokers. Fehringer et al. however, found no correlation between smoking and RCT.

In the present study, a multivariate logistic regression was utilized to mainly understand whether group membership (PG vs. CG) has an impact on RCT. Other co-variables like smoking, sport, handedness and age were also included in the analysis. The rationale of including the aforementioned variables was that they were, to our knowledge, the most commonly reported variables in literature hitherto to have an impact on the incidence of RCT in the normal population. Hence, we included them in the multivariable regression model to elucidate their interplay with RCT in our population.

In the multivariate logistic regression analysis, the correlation with the PG (OR 11.3; 95%-CI 4.868–26.182; p < .0001) greatly outweighed the correlations with smoking (OR 1.3; 95%-CI 0.608–2.596; p = 0.537). Smoking plays a role in increasing the risk for rotator cuff tear as described by Baumgarten et al. [33], but is not explaining the higher tear rate in the PG. The OR for the presence of RCT in the PG (OR = 11.289, 95%-CI 4.868–26.182; p < .0001) is much higher than the rate in the literature for smoking and the presence of RCT [33]. However, our analysis did not identify smoking (p = 0.537) as a risk factor (Table 9) in this pilot study.

### Limitations

The interpretation of our study results has several important limitations. First, CG was recruited by an advertisement in regional press. This recruitment method may not yield a true-cross-sectional representation of the general population and may result in a selection bias towards a better educated and healthier volunteers; however, this does not explain the 11-fold higher risk for RCT in the PG. Similarly, due to this method, groups were limited to male painters which also limits the generalizability of the results to both genders. Second, there were different proportions of smokers among the two groups. Smoking was not considered when the CG was recruited; thus, the impact of smoking as a co-variable may have differed between the two groups. Never the less, the multivariable logistic regression analysis did not identify smoking as a risk factor (OR = 1.3; 95%-CI 0.608–2.596; p = 0.537) (Table 9) in this study. Third, Co-morbidities (diabetes, obesity etc.) were not accounted for during the phase of study recruitment based on the results of publications like the one by Fehringer et al. [32] which suggested that comorbidities did not play a role in RCT. Thus, in our pilot study, the impact of co-morbidities stays unclear. Fourth, sample size could not be formally apriori calculated due to missing information about the primary endpoint. Hence, we chose the sample size to be 100 per group which is relatively small and the statistical power of the conclusion is not high. However, this was designed as a pilot study to investigate whether launching prospective long-term study is indicated. Fifth, goniometer measurements and clinical tests were only conducted once. Hence, we could not compute or report reliability in terms of clinical measurements. Finally, no individual stress analysis could be carried out among the painters, nor could we determine which type of occupational stress led to the wear and tear on the RC. Moreover, dose-dependent damage to the RC, i.e., a correlation between the time spent working as a painter and the presence of RCT, cannot be demonstrated on the basis of the analyzed data. This could only be determined in a prospective long-term study.

### Conclusion

This pilot study indicates that long-term occupational load on the shoulder of painters seems to be associated with an increased risk for developing RCT and clinical symptoms. Hence, this study may provide the first step to start developing preventative and rehabilitation programs to minimize disability in this population.

These programs should be similar to shoulder-protecting programs for athletes of over-head sports. They should include exercises to strengthen the rotator cuff muscles as well stabilize the glenohumeral joint. Finally, our study also gives impulse to launch prospective long-term studies to further investigate the intricacies and complex relationship between occurrence of RCT and working as a painter, or hand worker above shoulder level.
