# Correction: Could long-term overhead load in painters be associated with rotator cuff lesions? A pilot study

**DOI:** 10.1371/journal.pone.0218484

**Published:** 2019-06-12

**Authors:** Markus Loew, Sepehr Doustdar, Christoph Drath, Marc-André Weber, Thomas Bruckner, Felix Porschke, Patric Raiss, Marcus Schiltenwolf, Haidara Almansour, Michael Akbar

There is an error in affiliation 4 for author Marc-André Weber. The correct affiliation 4 is: Institute of Diagnostic and Interventional Radiology, Pediatric Radiology and Neuroradiology, University Medical Center Rostock, Rostock, Germany.
